# Weight loss and risk reduction of obesity-related outcomes in 0.5 million people: evidence from a UK primary care database

**DOI:** 10.1038/s41366-021-00788-4

**Published:** 2021-03-03

**Authors:** Christiane Lundegaard Haase, Sandra Lopes, Anne Helene Olsen, Altynai Satylganova, Volker Schnecke, Phil McEwan

**Affiliations:** 1 Novo Nordisk A/S, Søborg, Denmark; 2 Health Economics and Outcomes Research (HEOR) Ltd, Cardiff, UK

**Keywords:** Risk factors, Obesity

## Abstract

High body mass index (BMI) is known to be associated with various conditions, including type 2 diabetes (T2D), osteoarthritis, cardiovascular disease (CVD) and sleep apnoea; however, the impact of intentional weight loss on the risk of these and other outcomes is not well quantified. We examined the effect of weight loss on ten selected outcomes in a population from the UK Clinical Practice Research Datalink (CPRD) GOLD database. Included individuals were >18 years old at the index date (first BMI value between January 2001 and December 2010). They were categorised by their weight pattern between year 1 post-index and year 4 post-index (baseline period) as having stable weight (−5% to +5%) or weight loss (−25% to −10%, plus evidence of intervention or dietary advice to confirm intention to lose weight). For inclusion, individuals also required a BMI of 25.0–50.0 kg/m^2^ at the start of the follow-up period, during which the occurrence of ten obesity-related outcomes was recorded. Cox proportional hazard models adjusted for BMI, comorbidities, age, sex and smoking status were used to estimate relative risks for weight loss compared with stable weight. Individuals in the weight-loss cohort had median 13% weight loss. Assuming a BMI of 40 kg/m^2^ before weight loss, this resulted in risk reductions for T2D (41%), sleep apnoea (40%), hypertension (22%), dyslipidaemia (19%) and asthma (18%). Furthermore, weight loss was associated with additional benefits, with lower risk of T2D, chronic kidney disease, hypertension and dyslipidaemia compared with maintaining the corresponding stable lower BMI throughout the study. This study provides objective, real-world quantification of the effects of weight loss on selected outcomes, with the greatest benefits observed for the established CVD risk factors T2D, hypertension and dyslipidaemia.

## Introduction

Many of the clinical and economic [1] impacts of obesity are contributed by the presence of various chronic comorbidities, and the association between increasing body mass index (BMI) and the risk of these obesity-related outcomes has been extensively characterised. A report by the World Health Organization has summarised the impacts of obesity on multiple organ systems [2]; furthermore, observational studies have reported that various conditions, including type 2 diabetes (T2D) [3], sleep apnoea [4], osteoarthritis [3] and cardiovascular disease (CVD) [3, 5] are strongly associated with higher BMI. Increased mortality has been linked both to higher BMI [6] and to the presence of common obesity-related comorbidities [7]. A recent human development perspectives report by the World Bank Group [8] and a policy report from the Organisation for Economic Co-operation and Development [9] have emphasised these wide-ranging impacts of obesity.

Both the degree of overweight or obesity and the presence of comorbidities should be considered when identifying the best weight-management approach for each individual. Guidelines from the UK [10] and the USA [11] suggest that minimal weight loss of between 5 and 10% is sufficient to have a clinical impact on outcomes. Treatment approaches to achieve this include: dietary and lifestyle changes, such as increased physical activity; pharmacological intervention; and bariatric surgery for patients with severe obesity and comorbidities [12, 13]. There is evidence that these interventions affect the risk of obesity-related outcomes in addition to driving weight loss; indeed, a systematic review has shown that increased physical activity as an adjunct to dietary interventions resulted in increased weight loss as well as improvements in circulating lipid levels and blood pressure [14]. Data from randomised controlled trials have also demonstrated that weight-loss interventions, such as pharmacotherapy and lifestyle changes, can reduce the risk of obesity-related conditions including sleep apnoea [15–17] and delay the onset of T2D [18, 19]. In addition, a secondary analysis of data from the Intensive Diet and Exercise for Arthritis randomised controlled trial showed significant benefits in individuals with knee osteoarthritis who achieved weight loss of 10–20% [20]. Data from the Diabetes Prevention Program [21] and the Action for Health in Diabetes (Look AHEAD) study [22] indicate that lifestyle interventions that promote weight loss also have a beneficial effect on diabetes and CVD outcomes (in those losing >10% of their body weight in the first year), respectively. Furthermore, long-term follow-up data from the Swedish Obese Subjects study showed that bariatric surgery resulted in a significant reduction in cardiovascular (CV) mortality and occurrence of first-time (fatal and non-fatal) CV events [23], as well as incidence of T2D [24].

Although clinical improvements associated with weight loss have been observed across multiple studies, the exact benefits resulting from intentional weight loss remain challenging to quantify in clinical practice. To date, studies examining the effects of weight loss on prospective outcomes have been complicated by various limitations, particularly the difficulty that many individuals experience in maintaining weight reductions in the long term. Distinguishing between unintentional weight loss, which may result from chronic disease, and intentional weight loss can also be challenging, especially in retrospective real-world studies [25]. Previous studies have not investigated how the risk of obesity-related outcomes changes with intentional weight loss, in comparison with maintaining baseline weight.

Here, we have conducted a retrospective study using data from the UK Clinical Practice Research Datalink (CPRD) GOLD database to assess two research questions:

1. How does the risk of outcomes differ after weight loss compared with maintaining the corresponding stable higher BMI? (Objective 1).
2. How does the risk of outcomes differ after weight loss compared with maintaining the corresponding stable lower BMI? (Objective 2).

## Methods

### Data source

Data were extracted from CPRD GOLD [26], a database of anonymised primary care records, and merged with Hospital Episode Statistics linkage information and death registry data from the Office for National Statistics [27]. The CPRD is a widely used source of UK primary care data, which has been used in >2500 publications [28].

### Study design and patient population

**Fig. 1:**
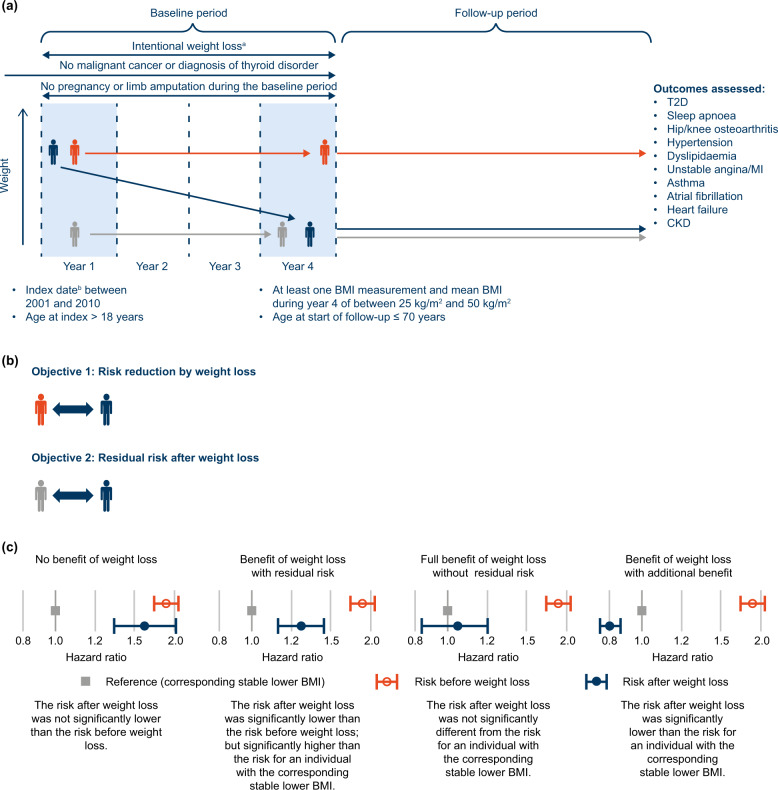
Study design and objectives. **a** Study design showing weight trajectories for the stable-weight (−5% to +5% BMI; orange), weight-loss cohorts (−25% to −10% BMI; blue) and corresponding stable lower BMI (grey). **b** Illustration of the comparisons made as part of objective 1 and objective 2 at the end of the follow-up period. **c** Example hazard ratio profiles (HRs and 95% CI) showing the comparative benefit patterns before weight loss (orange) with the risk after weight loss (blue) relative to the corresponding stable lower BMI (grey). ^a^Record indicating either a weight-loss diet, weight-loss drug prescription, or referral to a dietician or for bariatric surgery during the baseline period, to confirm the intention to lose weight; ^b^Date of first BMI calculation. BMI body mass index, CI confidence interval, CKD chronic kidney disease, HR hazard ratio, MI myocardial infarction, T2D type 2 diabetes.

Index date for each included individual was defined as the date of the earliest BMI calculation between January 2001 and December 2010 (Fig. 1a), and marked the beginning of year 1 of the study. BMI calculations were made during the baseline period (years 1–4 after index date), and the incidence of obesity-related outcomes was captured during the subsequent follow-up period. Follow-up ended at the date of the first event, death, transfer-out or the last data collection for the corresponding practice (January 2020 at the latest).

For inclusion, adult individuals (>18 years old at index date and ≤70 years at start of follow-up) were required to have at least one BMI calculation during year 1 and year 4, and a mean BMI between 25 and 50 kg/m^2^ during year 4 after the index date. Based on the change in their mean BMI between year 1 and year 4, individuals were assigned to one of two cohorts: the stable-weight cohort (−5% to +5% BMI change) or the weight-loss cohort (−25% to −10% BMI change). Individuals with weight change outside the ranges for these cohorts were excluded. Individuals in the weight-loss cohort required a Read code in CPRD GOLD indicating either a weight-loss diet, weight-loss drug prescription, or referral to a dietician or for bariatric surgery during the baseline period, to confirm the intention to lose weight. To further ensure that the weight loss observed could be considered intentional, individuals with malignant cancer or a diagnosis of thyroid disorder before the start of follow-up, and those with a record of pregnancy or limb amputation during the baseline period, were excluded from the study.

### Exposure and outcomes

We assessed the risks before and after weight loss for ten obesity-related outcomes: T2D, sleep apnoea, hip/knee osteoarthritis, hypertension, dyslipidaemia, unstable angina/myocardial infarction (MI; composite endpoint), asthma, atrial fibrillation, heart failure and chronic kidney disease (CKD). These outcomes were selected to provide a broad range of conditions and events associated with obesity, which represent the cardiovascular, metabolic, endocrine, musculoskeletal, respiratory and renal systems [7, 8, 29]. Event dates were defined as the earliest record of a Read code in CPRD GOLD or an International Classification of Diseases-10 code in Hospital Episode Statistics and Office for National Statistics death records (Supplementary Tables 1 and 2). For hypertension and dyslipidaemia, the event date was either the date of the earliest anti-hypertensive or lipid-lowering drug prescription, respectively, or the date of the earliest diagnostic code, whichever occurred first. Obesity-related outcomes that occurred before the start of the follow-up period were captured as baseline comorbidities. To allow estimation of the time to first incident diagnosis or event of an outcome, separate models were developed for each of the ten outcomes and individuals with a baseline history of the outcome were excluded from the risk analysis for that outcome only.

### Data presentation and interpretation

Two comparisons were performed to assess the benefit of weight loss (Fig. 1b). We estimated the difference in the risk of developing obesity-related outcomes for an individual in the weight-loss cohort compared with an individual in the stable-weight cohort who maintained a baseline BMI that was either:

1. Identical to the year 1 BMI of an individual who lost weight (i.e., before weight loss; objective 1), or
2. Identical to the year 4 BMI of an individual who lost weight (i.e., after weight loss; objective 2).

Baseline characteristics are presented as median and interquartile range for continuous variables and as proportions (%) for categorical variables. Risks are expressed as hazard ratios (HRs) and 95% confidence intervals (CIs) relative to the stable BMI in year 1 (objective 1) or year 4 (objective 2). For objective 2, we split the results into three different BMI profiles that encompassed the BMI range included in the study to allow assessment of the residual risk after weight loss (based on the median value in the weight-loss cohort).

Profile 1: BMI before weight loss: 35.0 kg/m^2^; BMI after weight loss: 30.5 kg/m^2^.

Profile 2: BMI before weight loss: 40.0 kg/m^2^; BMI after weight loss: 34.8 kg/m^2^.

Profile 3: BMI before weight loss: 45.0 kg/m^2^; BMI after weight loss: 39.2 kg/m^2^.

The effect of weight loss on each outcome was classified as one of four scenarios (Fig. 1c).

### Statistical analyses

Cox proportional hazard models with calendar time as the underlying time variable were used to estimate the differences in risks between the stable-weight and weight-loss cohorts. The main covariates were a categorical variable indicating the cohort (stable-weight/weight-loss), the BMI at the start of the follow-up period (i.e., BMI during year 4 of the baseline period), a quadratic term for the BMI, and an interaction term between the BMI and the cohort indicator. All models were adjusted for age, sex and smoking (never/ever). Four binary covariates were used to describe the prevalence of T2D, hypertension or dyslipidaemia, or the history of a CV event (transient ischaemic attack/stroke/unstable angina/MI), at the start of the follow-up period. A sensitivity analysis was also performed to compare the underlying covariate HRs in the whole study population and following the exclusion of individuals who had received sibutramine during the baseline period. All statistical analyses were performed using the R environment for statistical computing and visualisation (R Foundation for Statistical Computing; version 3.6.2).

## Results

### Study population and baseline characteristics

In total, 902,341 individuals met the inclusion criteria for this study, of whom 523,138 (58.0%) met the criteria for the stable-weight cohort and 76,110 (8.4%) met the criteria for the weight-loss cohort. Of those who met the weight-loss cohort criteria, 48,823 (64.1%) had evidence of an intention to lose weight. The overall study population included 571,961 individuals, of whom 523,138 were in the stable-weight cohort and 48,823 were in the weight-loss cohort (Table 1). In total, 49.2% of the population were men and 44.5% had never smoked. The median age at the start of the follow-up period was 55 years and the median follow-up time was 6.3 years. In the stable-weight cohort, the median BMI was 29.9 kg/m^2^ during year 1 and 30.0 kg/m^2^ during year 4. The corresponding median BMIs in the weight-loss cohort were 35.3 kg/m^2^ and 30.4 kg/m^2^, respectively, representing a median weight loss of 13%. In the weight-loss cohort, 57.6% of individuals were given dietary advice at some point during the 4-year baseline period, 52.7% reported that they initiated a weight-loss diet, 27.0% were prescribed a weight-loss medication and 1.1% were referred for bariatric surgery (CPRD GOLD) or underwent bariatric surgery (Hospital Episode Statistics).

**Table 1:** Baseline characteristics and weight-loss interventions for the study cohort at the start of follow-up.

	Total	Stable weight	Weight loss
*N*	571,961	523,138	48,823
Sex, men (%)	281,144 (49.2)	263,043 (50.3)	18,101 (37.1)
Smoking, ever (%)	317,679 (55.5)	288,378 (55.1)	29,301 (60.0)
Median age, year (IQR)	55 (45, 63)	55 (45, 63)	54 (44, 63)
Median weight year 1, kg (IQR)	87.1 (77.0, 99.6)	86.0 (76.2, 98.0)	98.4 (87.0, 13.0)
Median BMI year 1, kg/m^2^ (IQR)	30.3 (27.5, 34.2)	29.9 (27.3, 33.5)	35.3 (32.0, 40.0)
Median BMI year 4, kg/m^2^ (IQR)	30.1 (27.4, 33.8)	30.0 (27.4, 33.7)	30.4 (27.5, 34.4)
Median weight change, % (IQR)	+0.3 (−2.4, 3.0)	+0.8 (−1.6, 3.0)	−12.5 (−15.3, −11.0)
Median follow-up, years (IQR)	6.3 (3.4, 9.2)	6.4 (3.4, 9.3)	5.7 (2.8, 8.3)
Weight-loss intervention			
Patient-initiated diet, *n* (%)	213,490 (37.3)	187,744 (35.9)	25,746 (52.7)
Dietary advice, *n* (%)	181,643 (31.8)	153,534 (29.3)	28,109 (57.6)
Weight-loss medication, *n* (%)	55,113 (9.6)	41,943 (8.0)	13,170 (27.0)
Bariatric surgery, *n* (%)	643 (0.11)	100 (0.02)	543 (1.11)

*BMI* body mass index, *IQR* interquartile range.

**Fig. 2:**
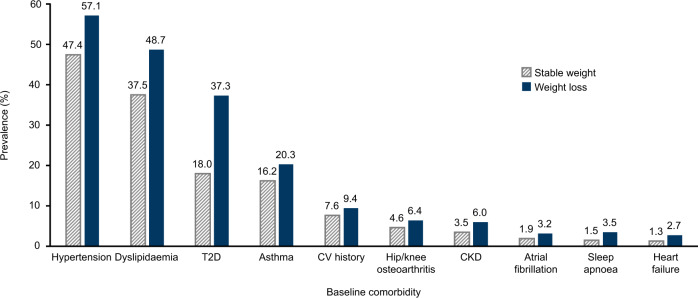
Prevalence of baseline comorbidities. Bar chart showing the prevalence of comorbidities at baseline (i.e., the start of the follow-up period) in the stable-weight and weight-loss cohorts. CKD chronic kidney disease, CV cardiovascular, T2D type 2 diabetes.

In the overall study population, the baseline comorbidities (observed at the start of the follow-up period) with the highest prevalence were hypertension (48.2%), dyslipidaemia (38.4%), T2D (19.7%) and asthma (16.5%). The prevalence of each comorbidity was higher in the weight-loss cohort than in the stable-weight cohort; the largest differences between cohorts were for sleep apnoea (2.3-fold difference between cohorts), heart failure (2.1-fold difference) and T2D (2.1-fold difference; Fig. 2).

When examining changes in body weight during the follow-up period, we observed a weight gain of ~5% in the weight-loss cohort and a weight gain of between 2 and 3% in the stable-weight cohort (Supplementary Fig. 1) during the first 2 years of follow up; however, during the rest of the follow-up period, there remained a stable long-term weight difference of ~10% between the cohorts, indicating that the distinction between the cohorts was maintained during the full study period.

### Risk reduction following weight loss (objective 1)

**Fig. 3:**
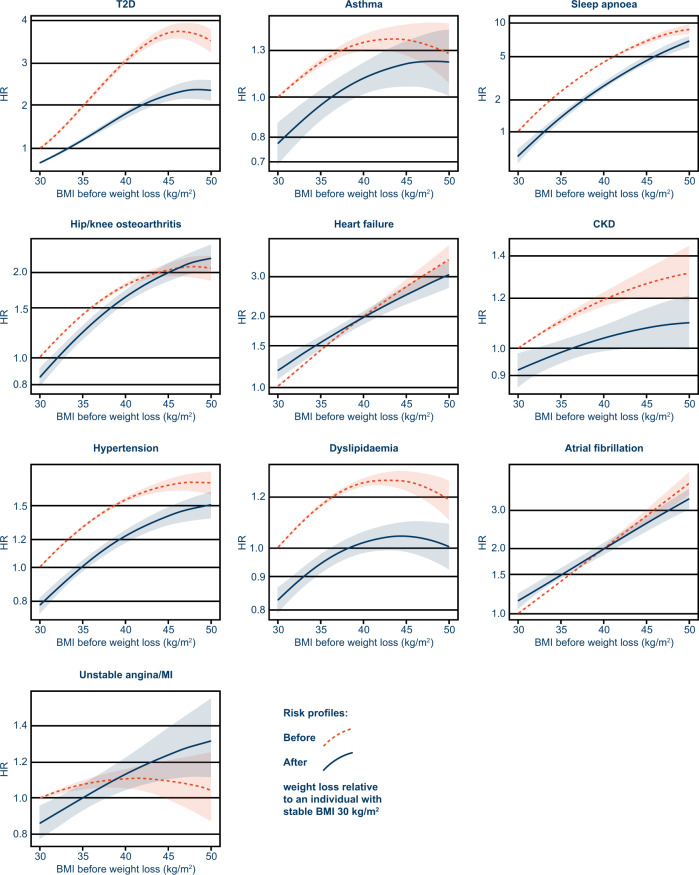
Risk profiles before and after weight loss for ten obesity-related outcomes (objective 1). Risk profiles and 95% CI (shaded area) showing the risk of outcomes before (dashed line) and after (solid line) weight loss. HRs are expressed relative to a stable BMI of 30 kg/m^2^. BMI body mass index, CI confidence interval, CKD chronic kidney disease, HR hazard ratio, MI myocardial infarction, T2D type 2 diabetes.

Figure 3 shows the risks for developing the ten obesity-related outcomes before and after weight loss for different BMIs at index, relative to an individual with a stable BMI of 30 kg/m^2^. The HRs and 95% CIs for these plots are presented in Supplementary Table 3. At index BMI 40 kg/m^2^, the greatest relative risk reductions with median 13% weight loss were observed for T2D and sleep apnoea (41% and 40%, respectively), followed by hypertension (22%), dyslipidaemia (19%) and asthma (18%).

HRs were estimated for all included covariates, allowing us to quantify changes in outcome risk with increasing baseline BMI or age. A 1-unit increase in BMI was associated with increases in relative risk for the development of sleep apnoea (HR [95% CI]: 1.20 [1.19–1.21]) and T2D (1.17 [1.17–1.18]; Supplementary Fig. 2). Similarly, a 1-year increase in baseline age was associated with increases in relative risk of developing atrial fibrillation (HR [95% CI]: 1.10 [1.10–1.10]), CKD (1.09 [1.09–1.09]) and heart failure (1.08 [1.07–1.08]; Supplementary Fig. 2).

### Assessment of residual risk after weight loss (objective 2)

**Fig. 4:**
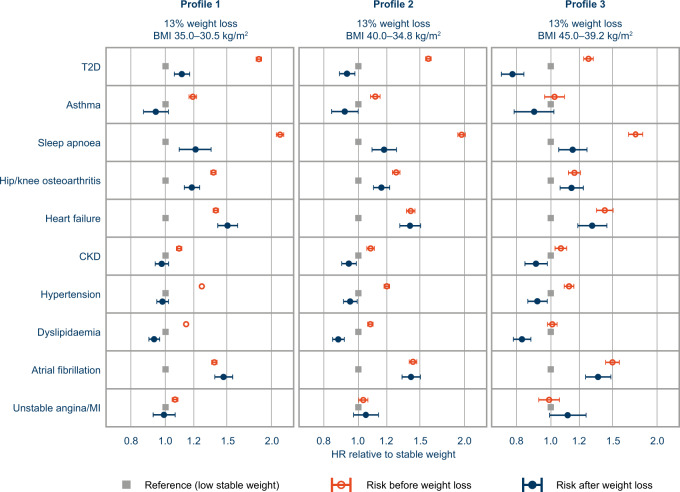
Risk profile for median 13% weight loss for ten obesity-related outcomes (objective 2). Changes in outcome risks are plotted as the risk before (orange open circles) and after (blue closed circles) weight loss relative to the corresponding stable lower BMI (grey squares) for each BMI profile. BMI body mass index, CKD chronic kidney disease, HR hazard ratio, MI myocardial infarction, T2D type 2 diabetes.

Figure 4 shows the benefit profiles associated with median weight loss of 13% for three BMI profiles, showing the risks of outcomes after weight loss compared with the stable higher and lower BMIs in each profile. The HRs and 95% CIs for these plots are presented in Supplementary Table 4 and a summary of the benefit scenarios (Fig. 1c) is given in Supplementary Table 5.

In profile 1 (Fig. 4), the risk of T2D before weight loss at BMI 35.0 kg/m^2^ was 84% higher than the risk at BMI 30.5 kg/m^2^ after weight loss, representing a considerable reduction in risk. However, the risk for an individual who had lost weight was still 11% higher than the risk for an individual who had maintained a stable BMI of 30.5 kg/m^2^. For BMI profiles 2 and 3 (Fig. 4), the risk of T2D after weight loss was lower than the risk for individuals with the corresponding stable lower BMI (HR [95% CI]: 0.93 [0.89–0.98] and 0.78 [0.73–0.84], respectively). This suggested that weight loss confers additional benefit, reducing the risk of developing T2D to below the level for an individual who had maintained the corresponding lower stable BMI.

Similarly, for hypertension, dyslipidaemia and CKD, weight loss was associated with additional benefits, compared with maintaining the corresponding lower stable BMI, across all BMI profiles. For hypertension and CKD, the benefit was greatest in BMI profiles 2 and 3 (Fig. 4). Similar results were observed for asthma in BMI profiles 1 and 2; however, results in BMI profile 3 (Fig. 4) were inconclusive.

Sleep apnoea was associated with the highest relative risks before weight loss, with HRs of 2.1, 2.0 and 1.8, respectively, for the three BMI profiles. For all profiles, median 13% weight loss was associated with risk reductions for sleep apnoea, but always with residual risk compared with the corresponding stable lower BMIs. Similar outcomes were observed for hip/knee osteoarthritis in BMI profiles 1 and 2, but no benefit of weight loss was observed in BMI profile 3 (Fig. 4). The results for the CV outcomes (heart failure, atrial fibrillation, unstable angina/MI) showed no clear difference in risk for weight loss compared with maintaining a high stable BMI, and in the case of unstable angina/MI did not show a difference compared with maintaining the corresponding stable lower BMI. This was consistent across the BMI profiles (Fig. 4).

### Sensitivity analysis following exclusion of individuals who had received sibutramine

When the covariate HRs for the overall study population (Supplementary Table 6) were compared to those generated following the exclusion of individuals who had received sibutramine (Supplementary Table 7), similar results were observed.

## Discussion

In this study, we estimated how the risks of ten obesity-related outcomes change in response to weight loss of 13%. The greatest benefits of weight loss were observed for outcomes known to be strongly associated with BMI: T2D, sleep apnoea, hypertension and dyslipidaemia [25]. These results support the findings of previous studies showing that moderate weight loss can reduce blood pressure, T2D biomarkers (fasting glucose and insulin levels, glycated haemoglobin), circulating lipids and other CVD risk biomarkers [18, 30–35]. We also found that the risks associated with sleep apnoea and hip/knee osteoarthritis were reduced after weight loss, but with a residual risk. Our findings for the CV outcomes (heart failure, atrial fibrillation, unstable angina/MI) were inconclusive.

We made an important observation that median 13% weight loss was associated with significant additional benefits for certain outcomes, notably T2D, CKD, hypertension and dyslipidaemia, compared with maintaining the corresponding stable lower BMI. One explanation is that weight loss may have been conferred by metabolic benefits, which contributed to some of the additional benefits. Although changes in lifestyle could not be captured in our analysis, we found that fewer than 30% of those in the weight-loss cohort had an initial referral for weight-loss medication or bariatric surgery, indicating that the weight loss in the remainder of the cohort was achieved without these interventions. Therefore, lifestyle changes might also be an explanation for the apparent additional benefit of weight loss.

We did not observe a clear reduction in the risk of heart failure, atrial fibrillation or unstable angina/MI after weight loss, suggesting that the duration of follow-up may not have been adequate to capture changes in the incidence of these events. With longer follow-up, the reductions in the occurrence of known CVD risk factors (T2D, dyslipidaemia, hypertension, CKD) that we observed in our study may have driven a detectable reduction in these CV outcomes. Furthermore, a relatively higher proportion of individuals in the weight-loss cohort had comorbidities at baseline, which may have resulted in a higher risk of CV outcomes, but may also have been an impetus for weight loss, confounding comparisons with the stable-weight cohort. An additional consideration is that some changes occurring over a significant time period before diseases become symptomatic, such as cardiac remodelling associated with heart failure [36], may not be reversed by weight loss.

To our knowledge, this is the first study to assess, in a single real-world population, the differential impact of intentional weight loss on a range of obesity-related outcomes, for different BMI profiles. A major strength of our study design was that the requirement for a record of weight-loss intervention or referral during the baseline period, and the exclusion of patients with evidence of conditions causing non-intentional weight loss, enabled us to restrict our analyses to those who intended to lose weight. Therefore, by linking our analyses to treatment approaches used to achieve weight loss, we have generated outcome risks observed across BMI profiles that can be used to inform risk stratification in clinical practice. Our results also have the potential to be used in future cost-effectiveness analyses of weight-loss interventions. A further strength is that we examined weight loss over a period of several years, but used mean BMI between year 1 and year 4 to characterise weight change, helping to mitigate the impact of temporary fluctuations in body weight. This timeframe also permitted flexibility in capturing valid weight measurements, allowing us to maximise the number of patients eligible for inclusion.

Due to the retrospective, observational nature of this analysis, the study is unable to provide conclusive evidence of the causative nature of the observations. It is likely that some factors contributing to weight loss were not captured, meaning that the causes of individuals’ weight loss could not always be fully elucidated. Similarly, comorbidities that were not recorded in CPRD GOLD or not captured at baseline may have contributed to the incidence of particular outcomes during follow-up. Our results may also have been affected by changes in prescribing practices during the study period. Two weight-loss drugs included as evidence of intention to lose weight during the baseline period, sibutramine and rimonabant, have since been withdrawn from the market [37], due to CV and psychiatric side effects, respectively. Therefore, these medications may have been discontinued prematurely during the study or may have contributed to the incidence of CV outcomes. However, a sensitivity analysis excluding patients on sibutramine produced similar covariate HRs and similar results to the main analyses, suggesting that this did not have a strong effect on our study. Another limitation of our study design was imposed by the need for delineation between individuals with stable weight and those with weight loss of 10% or more, to distinguish between risks for these two groups. This allowed the study to explore the effects of weight loss that exceeded 10% but meant that individuals in CPRD GOLD with weight loss between 5 and 10% were excluded.

In addition to the reduced symptomatic burden and improved health-related quality of life associated with weight loss, reducing the frequency of obesity-related outcomes is likely to alleviate the economic impact of the disease. T2D accounts for a large proportion of obesity-related healthcare costs [38], which increase over time and with disease severity. Therefore, these costs can be partially mitigated by early investments in strategies to prevent such comorbidities, such as weight-loss interventions [39]. In our analyses, median 13% weight loss was associated with significant additional benefits in terms of T2D risk, suggesting that intentional weight loss could result in substantial healthcare and economic savings associated with T2D alone. The additional benefits that we observed for CKD, hypertension and dyslipidaemia would also be expected to bring cost savings; however, a further analysis would be required to assess this possibility and to quantify the number needed to treat.

Our results have revealed disparities in the benefits of intentional weight loss depending on the outcome being examined, and future analyses should seek to assess the potential impact of other important factors on such observations. One highly relevant area of study would be the socioeconomic and lifestyle factors that may impact weight loss. Furthermore, as we observed additional benefits of weight loss associated with some outcomes (T2D, CKD, hypertension and dyslipidaemia) during the follow-up period, it would be of interest to assess how these patterns vary according to baseline characteristics including age, sex and comorbidity status.

This study provides objective quantification of the benefit of weight loss for relevant outcomes in a primary care setting, and substantiates the results of previous studies. The greatest benefits were observed for established CVD risk factors (T2D, hypertension and dyslipidaemia), CKD and sleep apnoea. Our results highlight the potential wider physical and healthcare benefits of weight loss and, by taking into account different BMI profiles, demographic characteristics and comorbidities, have broad relevance to inform treatment decisions made in clinical practice.

## Supplementary information

Supplementary Figure 1. Mean relative weight (with 95% CI; shaded area) for individuals in the stable-weight or weight-loss cohorts.

Supplementary Figure 2. Associations between outcome risk and a one-unit increase in baseline BMI (X-axis) or age (Y-axis).

Supplementary Tables 1 and 2. Disease classification codes.

Supplementary Table 3. Outcome risk before and after median 13% weight loss starting at BMI 35.0 kg/m^2^, 40.0 kg/m^2^ and 45.0 kg/m^2^ relative to a stable BMI of 30 kg/m^2^ (objective 1; Figure 3).

Supplementary Table 4. Outcome risk change associated with median 13% weight loss starting at BMI 35.0 kg/m^2^, 40.0 kg/m^2^ and 45.0 kg/m^2^ relative to the corresponding stable lower BMI.

Supplementary Table 5. Benefit of weight-loss scenarios across outcomes and BMI profiles.

Supplementary Table 6. Covariate hazard ratios (95% CI) in the whole study population.

Supplementary Table 7. Covariate hazard ratios (95% CI) for the study population following exclusion of individuals who received sibutramine.
