# Supplementary material for: Weight loss and risk reduction of obesity-related outcomes in 0.5 million people: evidence from a UK primary care database
Source: Int J Obes (Lond). 2021 Mar 3;45(6):1249–58. doi: 10.1038/s41366-021-00788-4 (PMC8159734; doi:10.1038/s41366-021-00788-4)

**Supplementary Figure 1. Mean relative weight (with 95% CI; shaded area) for individuals in the stable-weight or weight-loss cohorts.** The numbers above the symbols reflect the number of individuals with available data and the proportion who had their BMI calculated at each timepoint. ^a^Date of first BMI calculation. BMI, body mass index; CI, confidence interval.


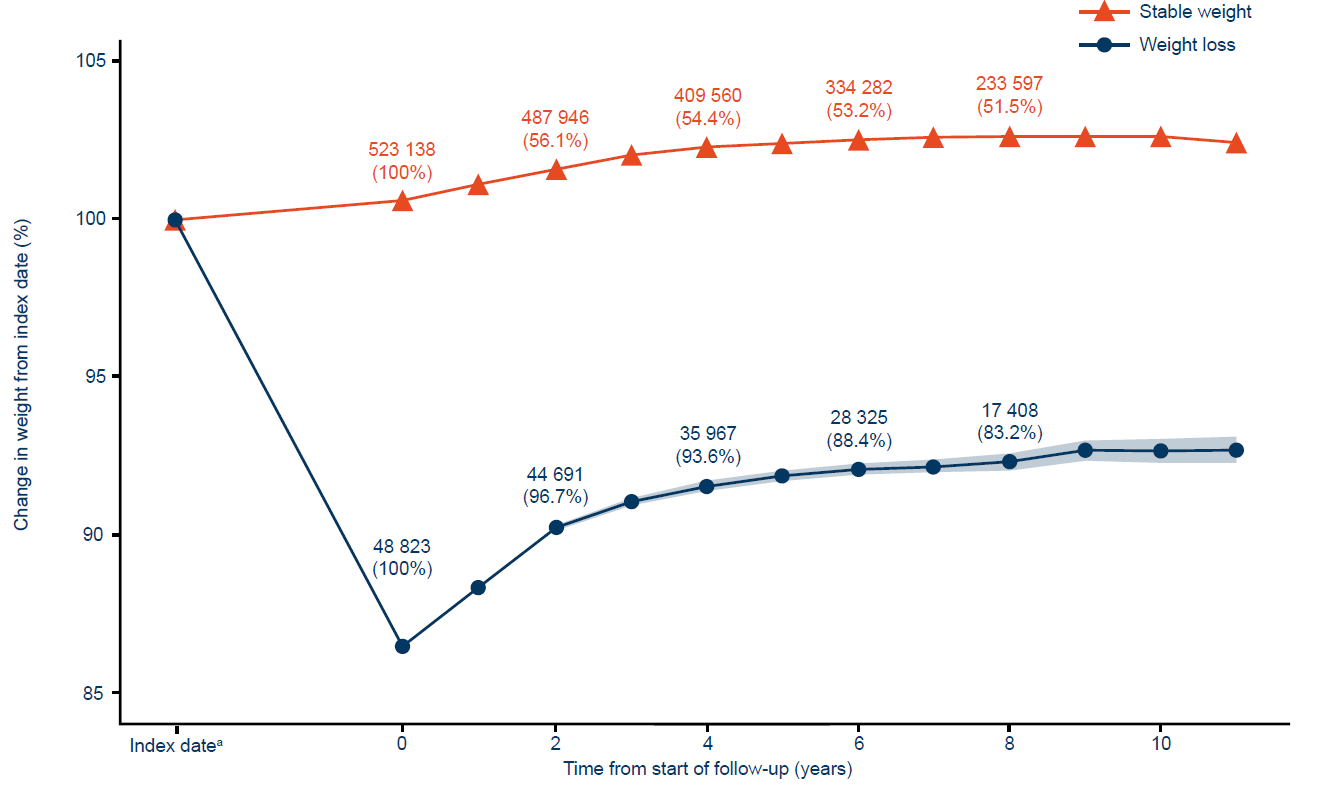

Supplement: Supplementary file 1 — Supplementary Figure 1. Mean relative weight (with 95% CI; shaded area) for individuals in the stable-weight or weight-loss cohorts. [file 41366_2021_788_MOESM1_ESM.docx]
