# Supplementary material for: Weight loss and risk reduction of obesity-related outcomes in 0.5 million people: evidence from a UK primary care database
Source: Int J Obes (Lond). 2021 Mar 3;45(6):1249–58. doi: 10.1038/s41366-021-00788-4 (PMC8159734; doi:10.1038/s41366-021-00788-4)

Supplementary Figure 2. Associations between outcome risk and a one-unit increase in baseline BMI (X-axis) or age (Y-axis). BMI, body mass index; CKD, chronic kidney disease; MI, myocardial infarction.


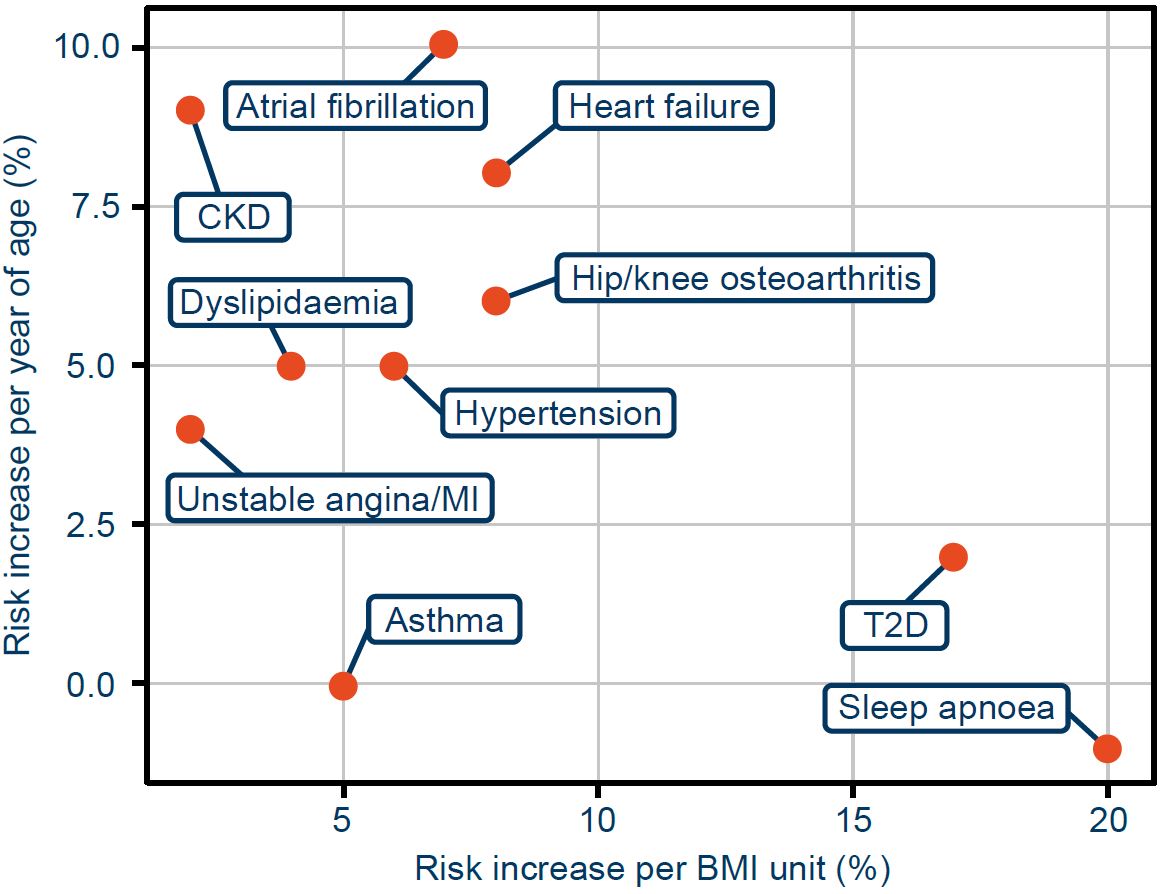

Supplement: Supplementary file 2 — Supplementary Figure 2. Associations between outcome risk and a one-unit increase in baseline BMI (X-axis) or age (Y-axis). [file 41366_2021_788_MOESM2_ESM.docx]
