# Supplementary material for: Weight loss and risk reduction of obesity-related outcomes in 0.5 million people: evidence from a UK primary care database
Source: Int J Obes (Lond). 2021 Mar 3;45(6):1249–58. doi: 10.1038/s41366-021-00788-4 (PMC8159734; doi:10.1038/s41366-021-00788-4)
Supplement: Supplementary file 4 — Supplementary Table 3. Outcome risk before and after median 13% weight loss starting at BMI 35.0 kg/m2, 40.0 kg/m2 and 45.0 kg/m2 relative to a stable BMI of 30 kg/m2 (objective 1; Figure 3). [file 41366_2021_788_MOESM4_ESM.docx]

**Supplementary Table 3. Outcome risk before and after median 13% weight loss starting at BMI 35.0 kg/m^2^, 40.0 kg/m^2^ and 45.0 kg/m^2^ relative to a stable BMI of 30 kg/m^2^ (objective 1; Figure 3).**

| **Outcome** | **BMI before weight loss (kg/m^2^)** | **BMI after weight loss (kg/m^2^)** | **HRs relative to a stable BMI of 30 kg/m^2a^** | | **Relative risk reduction (%)** |
| --- | --- | --- | --- | --- | --- |
|  |  |  | **Risk before weight loss, HR (95% CI)** | **Risk after weight loss, HR (95% CI)** |  |
| **T2D** | 35 | 30.5 | 1.97 (1.95–2.00) | 1.20 (1.14–1.26) | 39.3 |
|  | 40 | 34.8 | 3.05 (2.99–3.12) | 1.80 (1.71–1.90) | 41.0 |
|  | 45 | 39.2 | 3.70 (3.56–3.86) | 2.26 (2.10–2.43) | 39.1 |
| **Asthma** | 35 | 30.5 | 1.22 (1.19–1.25) | 0.96 (0.89–1.04) | 21.2 |
|  | 40 | 34.8 | 1.36 (1.30–1.42) | 1.11 (1.02–1.22) | 17.8 |
|  | 45 | 39.2 | 1.38 (1.26–1.50) | 1.21 (1.06–1.37) | 12.4 |
| **Sleep apnoea** | 35 | 30.5 | 2.29 (2.23–2.36) | 1.32 (1.19–1.46) | 42.6 |
|  | 40 | 34.8 | 4.37 (4.18–4.57) | 2.64 (2.42–2.88) | 39.6 |
|  | 45 | 39.2 | 6.93 (6.48–7.41) | 4.61 (4.18–5.08) | 33.5 |
| **Hip/knee osteoarthritis** | 35 | 30.5 | 1.42 (1.40–1.44) | 1.23 (1.17–1.30) | 13.1 |
|  | 40 | 34.8 | 1.80 (1.75–1.85) | 1.64 (1.55–1.73) | 8.9 |
|  | 45 | 39.2 | 2.04 (1.93–2.16) | 2.00 (1.86–2.16) | 1.9 |
| **Heart failure** | 35 | 30.5 | 1.43 (1.40–1.47) | 1.55 (1.45–1.66) | –8.0 |
|  | 40 | 34.8 | 2.00 (1.92–2.08) | 1.99 (1.85–2.13) | 0.6 |
|  | 45 | 39.2 | 2.70 (2.50–2.92) | 2.49 (2.26–2.75) | 7.9 |
| **CKD** | 35 | 30.5 | 1.10 (1.09–1.12) | 0.99 (0.94–1.03) | 10.6 |
|  | 40 | 34.8 | 1.19 (1.16–1.23) | 1.04 (0.99–1.09) | 13.1 |
|  | 45 | 39.2 | 1.27 (1.20–1.34) | 1.08 (1.00–1.15) | 15.0 |
| **Hypertension** | 35 | 30.5 | 1.30 (1.29–1.32) | 1.01 (0.97–1.04) | 22.7 |
|  | 40 | 34.8 | 1.56 (1.53–1.59) | 1.23 (1.18–1.28) | 21.5 |
|  | 45 | 39.2 | 1.72 (1.65–1.79) | 1.40 (1.32–1.49) | 18.5 |
| **Dyslipidaemia** | 35 | 30.5 | 1.16 (1.15–1.17) | 0.95 (0.92–0.98) | 18.7 |
|  | 40 | 34.8 | 1.26 (1.24–1.28) | 1.02 (0.98–1.06) | 19.0 |
|  | 45 | 39.2 | 1.27 (1.22–1.31) | 1.04 (0.98–1.10) | 17.9 |
| **Atrial fibrillation** | 35 | 30.5 | 1.42 (1.39–1.45) | 1.51 (1.42–1.60) | –6.1 |
|  | 40 | 34.8 | 2.01 (1.95–2.08) | 1.98 (1.86–2.10) | 1.6 |
|  | 45 | 39.2 | 2.84 (2.67–3.03) | 2.60 (2.39–2.82) | 8.7 |
| **Unstable angina/MI** | 35 | 30.5 | 1.07 (1.05–1.10) | 1.00 (0.93–1.08) | 6.8 |
|  | 40 | 34.8 | 1.11 (1.06–1.16) | 1.13 (1.04–1.22) | –2.1 |
|  | 45 | 39.2 | 1.10 (1.00–1.21) | 1.24 (1.10–1.39) | –12.9 |

^a^HRs are presented relative to the outcome risk for an individual with stable BMI of 30.0 kg/m^2^.

BMI, body mass index; CI, confidence interval; CKD, chronic kidney disease; HR, hazard ratio; MI, myocardial infarction; T2D, type 2 diabetes.
