# Supplementary material for: Weight loss and risk reduction of obesity-related outcomes in 0.5 million people: evidence from a UK primary care database
Source: Int J Obes (Lond). 2021 Mar 3;45(6):1249–58. doi: 10.1038/s41366-021-00788-4 (PMC8159734; doi:10.1038/s41366-021-00788-4)
Supplement: Supplementary file 5 — Supplementary Table 4. Outcome risk change associated with median 13% weight loss starting at BMI 35.0 kg/m2, 40.0 kg/m2 and 45.0 kg/m2 relative to the corresponding stable lower BMI. [file 41366_2021_788_MOESM5_ESM.docx]

**Supplementary Table 4. Outcome risk change associated with median 13% weight loss starting at BMI 35.0 kg/m^2^, 40.0 kg/m^2^ and 45.0 kg/m^2^ relative to the corresponding stable lower BMI (objective 2; Figure 4).**

| **Outcome** | **BMI before weight loss (kg/m^2^)** | **BMI after weight loss (kg/m^2^)** | | **HRs relative to corresponding stable lower BMI^a^** | |
| --- | --- | --- | --- | --- | --- |
|  |  |  |  | **Risk before weight loss, HR (95% CI)** | **Risk after weight loss, HR (95% CI)** |
| **T2D** | 35 | 30.5 | | 1.84 (1.82–1.86) | 1.11 (1.06–1.17) |
|  | 40 | 34.8 | | 1.58 (1.56–1.60) | 0.93 (0.89–0.98) |
|  | 45 | 39.2 | | 1.28 (1.25–1.32) | 0.78 (0.73–0.84) |
| **Asthma** | 35 | | 30.5 | 1.19 (1.17–1.22) | 0.94 (0.87–1.02) |
|  | 40 | | 34.8 | 1.12 (1.09–1.15) | 0.92 (0.84–1.00) |
|  | 45 | | 39.2 | 1.03 (0.97–1.09) | 0.90 (0.79–1.02) |
| **Sleep apnoea** | 35 | | 30.5 | 2.11 (2.06–2.17) | 1.21 (1.09–1.34) |
|  | 40 | | 34.8 | 1.96 (1.92–2.01) | 1.19 (1.10–1.29) |
|  | 45 | | 39.2 | 1.75 (1.67–1.83) | 1.16 (1.06–1.28) |
| **Hip/knee osteoarthritis** | 35 | | 30.5 | 1.37 (1.35–1.39) | 1.19 (1.13–1.25) |
|  | 40 | | 34.8 | 1.28 (1.26–1.31) | 1.17 (1.11–1.23) |
|  | 45 | | 39.2 | 1.17 (1.13–1.22) | 1.15 (1.07–1.24) |
| **Heart failure** | 35 | | 30.5 | 1.39 (1.36–1.42) | 1.50 (1.40–1.60) |
|  | 40 | | 34.8 | 1.41 (1.38–1.45) | 1.40 (1.31–1.50) |
|  | 45 | | 39.2 | 1.43 (1.35–1.51) | 1.32 (1.19–1.45) |
| **CKD** | 35 | | 30.5 | 1.09 (1.08–1.11) | 0.98 (0.94–1.02) |
|  | 40 | | 34.8 | 1.09 (1.07–1.11) | 0.94 (0.90–0.99) |
|  | 45 | | 39.2 | 1.07 (1.03–1.11) | 0.91 (0.85–0.98) |
| **Hypertension** | 35 | | 30.5 | 1.27 (1.26–1.28) | 0.98 (0.95–1.02) |
|  | 40 | | 34.8 | 1.21 (1.19–1.23) | 0.95 (0.91–0.99) |
|  | 45 | | 39.2 | 1.13 (1.10–1.16) | 0.92 (0.87–0.98) |
| **Dyslipidaemia** | 35 | | 30.5 | 1.14 (1.14–1.15) | 0.93 (0.90–0.96) |
|  | 40 | | 34.8 | 1.09 (1.07–1.10) | 0.88 (0.85–0.91) |
|  | 45 | | 39.2 | 1.01 (0.99–1.04) | 0.83 (0.79–0.88) |
| **Atrial fibrillation** | 35 | | 30.5 | 1.38 (1.35–1.40) | 1.46 (1.38–1.55) |
|  | 40 | | 34.8 | 1.44 (1.41–1.47) | 1.41 (1.33–1.50) |
|  | 45 | | 39.2 | 1.50 (1.43–1.57) | 1.37 (1.26–1.49) |
| **Unstable angina/MI** | 35 | | 30.5 | 1.06 (1.04–1.09) | 0.99 (0.92–1.07) |
|  | 40 | | 34.8 | 1.03 (1.00–1.07) | 1.05 (0.97–1.14) |
|  | 45 | | 39.2 | 0.99 (0.93–1.06) | 1.12 (1.00–1.26) |

^a^HRs are presented relative to individuals with stable BMI of 30.5 kg/m^2^, 34.8 kg/m^2^ or 39.2 kg/m^2^, respectively (HR = 1.0 [ref]).

BMI, body mass index; CI, confidence interval; CKD, chronic kidney disease; HR, hazard ratio; MI, myocardial infarction; T2D, type 2 diabetes.
