# Supplementary material for: Weight loss and risk reduction of obesity-related outcomes in 0.5 million people: evidence from a UK primary care database
Source: Int J Obes (Lond). 2021 Mar 3;45(6):1249–58. doi: 10.1038/s41366-021-00788-4 (PMC8159734; doi:10.1038/s41366-021-00788-4)
Supplement: Supplementary file 6 — Supplementary Table 5. Benefit of weight-loss scenarios across outcomes and BMI profiles. [file 41366_2021_788_MOESM6_ESM.docx]

**Supplementary Table 5. Benefit of weight-loss scenarios across outcomes and BMI profiles.**

| **Outcome** | **Median 13% weight loss from  BMI 35.0 kg/m^2^ to 30.5 kg/m^2^** | **Median 13% weight loss from  BMI 40.0 kg/m^2^ to 34.8 kg/m^2^** | **Median 13% weight loss from  BMI 45.0 kg/m^2^ to 39.2 kg/m^2^** |
| --- | --- | --- | --- |
| **T2D** | Benefit of weight loss with residual risk | Benefit of weight loss with additional benefit | Benefit of weight loss with additional benefit |
| **Dyslipidaemia** | Benefit of weight loss with additional benefit | Benefit of weight loss with additional benefit | Benefit of weight loss with additional benefit |
| **Hypertension** | Full benefit of weight loss without residual risk | Benefit of weight loss with additional benefit | Benefit of weight loss with additional benefit |
| **CKD** | Full benefit of weight loss without residual risk | Benefit of weight loss with additional benefit | Benefit of weight loss with additional benefit |
| **Asthma** | Full benefit of weight loss without residual risk | Full benefit of weight loss without residual risk | No benefit of weight loss observed |
| **Sleep apnoea** | Benefit of weight loss with residual risk | Benefit of weight loss with residual risk | Benefit of weight loss with residual risk |
| **Hip/knee osteoarthritis** | Benefit of weight loss with residual risk | Benefit of weight loss with residual risk | No benefit of weight loss observed |
| **Atrial fibrillation** | No benefit of weight loss observed | No benefit of weight loss observed | No benefit of weight loss observed |
| **Heart failure** | No benefit of weight loss observed | No benefit of weight loss observed | No benefit of weight loss observed |
| **Unstable angina/MI** | No benefit of weight loss observed | No benefit of weight loss observed | No benefit of weight loss observed |

BMI, body mass index; CKD, chronic kidney disease; MI, myocardial infarction; T2D, type 2 diabetes.
