# Supplementary material for: Weight loss and risk reduction of obesity-related outcomes in 0.5 million people: evidence from a UK primary care database
Source: Int J Obes (Lond). 2021 Mar 3;45(6):1249–58. doi: 10.1038/s41366-021-00788-4 (PMC8159734; doi:10.1038/s41366-021-00788-4)
Supplement: Supplementary file 7 — Supplementary Table 6. Covariate hazard ratios (95% CI) in the whole study population. [file 41366_2021_788_MOESM7_ESM.docx]

**Supplementary Table 6.** **Covariate hazard ratios (95% CI) in the whole study population**

| **Outcome** | **Cohort Weight Loss** | **BMI** | **BMI^2^** | **Cohort:**  **BMI** | **Age** | **Sex (male)** | **Smoking (ever)** | **Comorbidities at start of follow-up period** | | | |
| --- | --- | --- | --- | --- | --- | --- | --- | --- | --- | --- | --- |
|  |  |  |  |  |  |  |  | **T2D** | **Hypertension** | **Dyslipidaemia** | **CV history** |
| **T2D** | 1.14 (1.08–1.20) | **1.17 (1.17–1.18)** | **0.995 (0.995–0.995)** | **0.960 (0.952–0.969)** | **1.02 (1.02–1.02)** | **1.47 (1.44–1.50)** | **1.22 (1.19–1.24)** |  | **1.44 (1.41–1.48)** | **1.42 (1.38–1.46)** | **1.09 (1.05–1.13)** |
| **Asthma** | 0.94 (0.87–1.02) | **1.05 (1.04–1.06)** | **0.998 (0.998–0.999)** | 0.995 (0.980–1.010) | 1.00 (1.00–1.00) | **0.67 (0.65–0.70)** | **1.20 (1.15–1.25)** | **0.74 (0.70–0.79)** | 1.01 (0.96–1.06) | **1.10 (1.05–1.16)** | 0.95 (0.88–1.03) |
| **Sleep apnoea** | 1.22 (1.09–1.35) | **1.20 (1.19–1.21)** | **0.996 (0.996–0.997)** | 0.995 (0.982–1.008) | **0.99 (0.99–0.99)** | **3.09 (2.94–3.25)** | **1.24 (1.19–1.30)** | **0.89 (0.84–0.94)** | **1.15 (1.09–1.22)** | **1.15 (1.09–1.22)** | 1.07 (0.99–1.16) |
| **Hip/knee osteoarthritis** | 1.19 (1.13–1.26) | **1.08 (1.08–1.09)** | **0.998 (0.997–0.998)** | 0.996 (0.987–1.005) | **1.06 (1.06–1.06)** | **0.83 (0.80–0.85)** | **0.96 (0.93–0.99)** | **0.82 (0.79–0.85)** | 1.00 (0.97–1.03) | 0.97 (0.94–1.00) | **0.93 (0.88–0.98)** |
| **Heart failure** | 1.51 (1.41–1.62) | **1.08 (1.07–1.09)** | 0.999 (0.999–1.000) | **0.985 (0.974–0.996)** | **1.08 (1.07–1.08)** | **1.51 (1.44–1.57)** | **1.47 (1.40–1.53)** | **1.52 (1.45–1.58)** | **1.60 (1.52–1.69)** | **1.14 (1.09–1.20)** | **2.54 (2.42–2.66)** |
| **CKD** | 0.98 (0.94–1.02) | **1.02 (1.02–1.03)** | 1.000 (0.999–1.000) | 0.992 (0.984–1.000) | **1.09 (1.09–1.09)** | **0.76 (0.74–0.78)** | **1.03 (1.01–1.06)** | **1.88 (1.83–1.93)** | **1.94 (1.88–2.01)** | **1.08 (1.05–1.11)** | **1.42 (1.38–1.47)** |
| **Hypertension** | 0.98 (0.95–1.02) | **1.06 (1.06–1.07)** | **0.998 (0.998–0.999)** | 0.993 (0.986–1.000) | **1.05 (1.05–1.05)** | **1.77 (1.74–1.80)** | **1.05 (1.04–1.07)** | **1.60 (1.56–1.64)** |  | **1.20 (1.17–1.22)** | **1.11 (1.06–1.17)** |
| **Dyslipidaemia** | 0.94 (0.90–0.97) | **1.04 (1.04–1.04)** | **0.999 (0.998–0.999)** | **0.987 (0.981–0.994)** | **1.05 (1.05–1.05)** | **1.45 (1.43–1.47)** | **1.24 (1.22–1.26)** | **2.46 (2.40–2.52)** | **1.51 (1.48–1.53)** |  | **1.34 (1.26–1.43)** |
| **Atrial fibrillation** | 1.46 (1.38–1.55) | **1.07 (1.07–1.08)** | 1.000 (0.999–1.000) | 0.993 (0.983–1.002) | **1.10 (1.10–1.10)** | **1.85 (1.78–1.91)** | **1.08 (1.05–1.12)** | **0.89 (0.86–0.92)** | **1.49 (1.43–1.55)** | 0.99 (0.95–1.03) | **1.36 (1.30–1.42)** |
| **Unstable angina/ MI** | 0.99 (0.91–1.06) | **1.02 (1.01–1.02)** | 0.999 (0.999–1.000) | **1.014 (1.001–1.028)** | **1.04 (1.03–1.04)** | **1.89 (1.81–1.97)** | **1.52 (1.46–1.59)** | **1.38 (1.32–1.45)** | **1.26 (1.20–1.32)** | **1.44 (1.38–1.51)** | **1.31 (1.21–1.43)** |

BMI, body mass index; CI, confidence interval; CKD, chronic kidney disease; CV, cardiovascular; MI, myocardial infarction; T2D type 2 diabetes.
