# Supplementary material for: Weight loss and risk reduction of obesity-related outcomes in 0.5 million people: evidence from a UK primary care database
Source: Int J Obes (Lond). 2021 Mar 3;45(6):1249–58. doi: 10.1038/s41366-021-00788-4 (PMC8159734; doi:10.1038/s41366-021-00788-4)
Supplement: Supplementary file 8 — Supplementary Table 7. Covariate hazard ratios (95% CI) for the study population following exclusion of individuals who received sibutramine. [file 41366_2021_788_MOESM8_ESM.docx]

**Supplementary Table 7.** **Covariate hazard ratios (95% CI) for the study population following exclusion of individuals who received sibutramine**

| **Outcome** | **Weight loss** | **BMI** | **BMI^2^** | **Weight loss:BMI** | **Age** | **Sex (male)** | **Smoking (ever)** | **Comorbidities at start of follow-up period** | | | |
| --- | --- | --- | --- | --- | --- | --- | --- | --- | --- | --- | --- |
|  |  |  |  |  |  |  |  | **T2D** | **Hypertension** | **Dyslipidaemia** | **CV history** |
| **T2D** | 1.15 (1.09–1.21) | 1.17 (1.17–1.18) | 0.995 (0.995–0.995) | 0.956 (0.947–0.966) | 1.02 (1.02–1.02) | 1.46 (1.43–1.50) | 1.21 (1.19–1.24) |  | 1.44 (1.41–1.48) | 1.42 (1.39–1.46) | 1.09 (1.05–1.13) |
| **Asthma** | 0.94 (0.86–1.02) | 1.05 (1.04–1.06) | 0.998 (0.998–0.999) | 0.993 (0.977–1.009) | 1.00 (1.00–1.00) | 0.68 (0.65–0.71) | 1.20 (1.15–1.25) | 0.74 (0.69–0.78) | 1.01 (0.96–1.06) | 1.11 (1.05–1.17) | 0.95 (0.87–1.03) |
| **Sleep apnoea** | 1.18 (1.05–1.32) | 1.20 (1.19–1.21) | 0.996 (0.996–0.997) | 0.996 (0.982–1.010) | 0.99 (0.99–0.99) | 3.16 (3.01–3.33) | 1.24 (1.18–1.30) | 0.89 (0.84–0.94) | 1.15 (1.09–1.22) | 1.15 (1.09–1.22) | 1.06 (0.98–1.15) |
| **Hip/knee osteoarthritis** | 1.18 (1.11–1.24) | 1.08 (1.08–1.09) | 0.998 (0.997–0.998) | 0.996 (0.986–1.005) | 1.06 (1.06–1.06) | 0.83 (0.81–0.85) | 0.96 (0.93–0.99) | 0.83 (0.80–0.86) | 1.00 (0.97–1.03) | 0.97 (0.94–1.00) | 0.93 (0.89–0.98) |
| **Heart failure** | 1.52 (1.41–1.63) | 1.08 (1.07–1.09) | 0.999 (0.999–1.000) | 0.982 (0.971–0.994) | 1.08 (1.07–1.08) | 1.51 (1.44–1.57) | 1.47 (1.41–1.54) | 1.51 (1.45–1.58) | 1.61 (1.52–1.70) | 1.14 (1.09–1.20) | 2.55 (2.43–2.67) |
| **CKD** | 0.98 (0.94–1.03) | 1.02 (1.02–1.03) | 1.000 (0.999–1.000) | 0.991 (0.982–0.999) | 1.09 (1.09–1.09) | 0.76 (0.74–0.78) | 1.03 (1.01–1.06) | 1.88 (1.83–1.93) | 1.94 (1.88–2.01) | 1.08 (1.05–1.11) | 1.42 (1.38–1.47) |
| **Hypertension** | 0.99 (0.95–1.03) | 1.06 (1.06–1.07) | 0.998 (0.998–0.999) | 0.992 (0.985–1.000) | 1.05 (1.05–1.05) | 1.77 (1.74–1.81) | 1.06 (1.04–1.07) | 1.60 (1.56–1.64) |  | 1.19 (1.17–1.22) | 1.11 (1.06–1.17) |
| **Dyslipidaemia** | 0.93 (0.90–0.97) | 1.04 (1.04–1.04) | 0.999 (0.998–0.999) | 0.985 (0.979–0.992) | 1.05 (1.05–1.05) | 1.45 (1.43–1.47) | 1.24 (1.22–1.26) | 2.44 (2.39–2.50) | 1.51 (1.48–1.54) |  | 1.35 (1.26–1.44) |
| **Atrial fibrillation** | 1.45 (1.37–1.54) | 1.07 (1.07–1.08) | 1.000 (0.999–1.000) | 0.994 (0.984–1.004) | 1.10 (1.10–1.10) | 1.84 (1.78–1.91) | 1.08 (1.05–1.12) | 0.89 (0.86–0.92) | 1.49 (1.43–1.56) | 0.99 (0.95–1.03) | 1.36 (1.30–1.42) |
| **Unstable angina / MI** | 0.99 (0.91–1.07) | 1.02 (1.01–1.02) | 0.999 (0.999–1.000) | 1.017 (1.003–1.031) | 1.04 (1.03–1.04) | 1.89 (1.81–1.98) | 1.52 (1.46–1.59) | 1.38 (1.32–1.44) | 1.26 (1.20–1.32) | 1.45 (1.38–1.52) | 1.31 (1.21–1.43) |

BMI, body mass index; CI, confidence interval; CKD, chronic kidney disease; CV, cardiovascular; MI, myocardial infarction; T2D type 2 diabetes.
